# A Critical Period for Postnatal Adaptive Plasticity in a Model of Motor Axon Miswiring

**DOI:** 10.1371/journal.pone.0123643

**Published:** 2015-04-13

**Authors:** Michaela S. Helmbrecht, Heidi Soellner, Maria A. Castiblanco-Urbina, Stefan Winzeck, Julia Sundermeier, Fabian J. Theis, Karim Fouad, Andrea B. Huber

**Affiliations:** 1 Institute of Developmental Genetics, Helmholtz-Zentrum München—German Research Center for Environmental Health (GmbH), Ingolstaedter Landstrasse 1, Neuherberg, Germany; 2 Institute for Computational Biology, Helmholtz-Zentrum München—German Research Center for Environmental Health (GmbH), Ingolstaedter Landstrasse 1, Neuherberg, Germany; 3 Department of Mathematics, Technische Universität München, Garching, Germany; 4 Faculty of Rehabilitation Medicine, University of Alberta, Edmonton, Canada; Virginia Tech Carilion Research Institute, UNITED STATES

## Abstract

The correct wiring of neuronal circuits is of crucial importance for precise neuromuscular functionality. Therefore, guidance cues provide tight spatiotemporal control of axon growth and guidance. Mice lacking the guidance cue Semaphorin 3F (Sema3F) display very specific axon wiring deficits of motor neurons in the medial aspect of the lateral motor column (LMCm). While these deficits have been investigated extensively during embryonic development, it remained unclear how *Sema3F* mutant mice cope with these errors postnatally. We therefore investigated whether these animals provide a suitable model for the exploration of adaptive plasticity in a system of miswired neuronal circuitry. We show that the embryonically developed wiring deficits in *Sema3F* mutants persist until adulthood. As a consequence, these mutants display impairments in motor coordination that improve during normal postnatal development, but never reach wildtype levels. These improvements in motor coordination were boosted to wildtype levels by housing the animals in an enriched environment starting at birth. In contrast, a delayed start of enriched environment housing, at 4 weeks after birth, did not similarly affect motor performance of *Sema3F* mutants. These results, which are corroborated by neuroanatomical analyses, suggest a critical period for adaptive plasticity in neuromuscular circuitry. Interestingly, the formation of perineuronal nets, which are known to close the critical period for plastic changes in other systems, was not altered between the different housing groups. However, we found significant changes in the number of excitatory synapses on limb innervating motor neurons. Thus, we propose that during the early postnatal phase, when perineuronal nets have not yet been formed around spinal motor neurons, housing in enriched environment conditions induces adaptive plasticity in the motor system by the formation of additional synaptic contacts, in order to compensate for coordination deficits.

## Introduction

During development, the wiring of neuronal circuits is tightly regulated by guidance cues and their receptors in order to allow for the correct functionality of the nervous system [1]. In the motor system of the brachial spinal cord, many guidance cues like ephrins and their Eph receptors [2, 3], GDNF and its receptor c-Ret [4], or signaling of secreted semaphorins with their neuropilin receptors [5], work together in order to guide the developing axons to their appropriate targets and lay the foundation for coordinated movements and motor control during postnatal life.

After development, the central nervous system is generally thought to be hardwired since injury or degeneration cannot be compensated and result in permanent damage. However, since the early work of Hubel and Wiesel there is evidence for special critical periods in which the central nervous system is still sensitive for external stimulation causing plastic rearrangements [6, 7]. Thus, in the motor system synapse elimination at neuromuscular junctions depends on neuronal activity during early postnatal live [8]. Furthermore, in the brain plastic rearrangements have been found in neuronal circuits of the visual and auditory cortex and also here these adaptive changes were only possible within specific sensitive periods [9–11]. Interestingly, external factors seem to play an important role during this process, since environmental enrichment was shown to have strong influences on adaptive plasticity in the brain [12–15]. Specific treadmill training and environmental enrichment were also found to have beneficial effects on neuronal plasticity after spinal cord injury [16–18].

Thus, investigations of structural plasticity in different neuronal circuits have defined a new picture of the mechanisms that allow the nervous system to adapt to changing conditions. However, in the spinal cord those studies have mainly focused on mechanisms of repair after injury. Since spinal cord injury models are hard to standardize even within the same laboratory and are usually accompanied by adverse secondary effects like scar formation and inflammation, we aimed to find a new model for the analysis of structural adaptive plasticity that does not depend on the extrinsic induction of an injury. In this respect the genetically induced axon miswiring in Semaphorin3F (*Sema3F*) knockout animals provides a very suitable model system, since it is very specific and affects exclusively motor neurons of the medial division of the lateral motor column (LMCm) [5, 19].

In the current study, we show that the developmental axon pathfinding deficits in *Sema3F* mutants result in impairments in motor coordination. By housing of the animals in enriched environment conditions we were able to induce plasticity in this model system, which are manifested by behavioral and neuroanatomical changes. Furthermore, we show evidence for a critical period for these adaptive changes since delayed exposure to enriched conditions did not reveal the same effects. Surprisingly, upon investigation of perineuronal nets, which have been shown to influence the closure of critical periods [20], we found no differences between normal and enriched environment housing. However, early postnatal exposure to an enriched environment induces synapse formation on miswired motor neurons that might be responsible for the induction of adaptive plasticity in the spinal motor system.

## Material and Methods

Mice were handled and housed according to the federal guidelines for the use and care of laboratory animals. All experimental procedure were approved by and conducted in adherence to the guidelines of the Regierung von Oberbayern.

Male *Sema3F* mice [19] in a C57BL/6 background were housed in groups of three per cage at a 12:12 light:dark cycle with food and water available ad libitum. Mice were housed under standard laboratory conditions in individually ventilated cages (IVCs, Biozone Ltd.). The enriched environment group was housed in large cages (37 cm x 21 cm x 18 cm) in groups of five animals per cage containing additional devices, that were changed on a weekly basis: Mouse Low Profile Wireless Running WheelR (Med Associates, Inc.), custom-made mini step ladder (10 cm x 5 cm x 5 cm with 19 rungs of 5 mm diameter each), wood-wool (5 g, Abedd) and metal objects for shelter.

### Behavior data acquisition and analysis

All behavioral data were measured by experimenters blinded to the genotype of the tested mice. Results were calculated as mean values ± SEM using Prism 5.0 software (GraphPad Software, La Jolla). Unpaired 2-tailed Student’s t test was used for single comparisons of normally distributed values, otherwise the Mann-Whitney test was applied. The effect of postnatal development of mice over time on the behavioral performance was assessed by 2-way repeated measures analysis of variance (ANOVA). A p-value for the interaction of genotype time less than 0.05 was set as statistical significance.

#### Open field

Mice were tested in the open field apparatus (Actimot Systems, TSE, Bad Homburg) in the dark to reduce possible effects of anxiety on gross locomotor behavior of rodents [21] during the light phase of the light:dark cycle for 20 min. Horizontal (distance travelled) and vertical locomotion (number of rearings) and average locomotor speed parameters were extracted from recordings of the automated video-tracking system (TSE Systems, GmbH). An additional cohort of *Sema3F* mice was tested for anxiety and explorative behavior at illumination levels of 300 lux in the corners and 350 lux in the middle of the test arena. The following parameters were analyzed individually for 3 areas (center, periphery and total arena): resting and permanence time, distance travelled, average speed of locomotion, percentage of time spent in the center, latency to first center entry and center enter frequency.

#### Gait analysis

For the analysis of specific gait parameters the Catwalk7.1 system (Noldus, Wageningen, Netherlands) was used [22]. The following settings were applied during data acquisition: Contrast (‰): 3990; Brightness x 0.001 V: -420; Pixel intensity threshold: 40; Pixel number threshold: 3. Animals were tested at the age of 9 weeks. From the 6 runs videotaped during data acquisition, the 3 best were chosen based on the following criteria: comparable walking speed in the different runs, a minimum of 3 complete step cycles and a straight and continuous walk without stoppings. Runs were pre-processed with an analysis pixel threshold of 25 to differentiate unspecific background from paw prints before paw classification. The pixel areas of each paw print were classified manually as right or left fore- or hindlimb. The following parameters were used for analysis: Forelimb base of support (mm), forelimb duty cycle (%) and stepping pattern.

#### Grid walk

Animals were tested at 4, 8 and 12 weeks of age. The time to cross a horizontal ladder (74 cm length) with irregularly spaced round metal rungs (1 mm diameter) as well as the number of paw placement errors by either missing the rung or slipping off the rung was averaged over 3 successive trials.

### Neuroanatomical analysis

Surgical procedures were carried out under aseptic conditions using i.p. Ketamine (0.1 mg/g), Xylazine (0.01 mg/g) anesthesia with Meloxicam (2 μg/g) as analgesic.

#### Retrograde labeling of dorsal and ventral forelimb motor pools

At 4 or 12 weeks of age Alexa-conjugated Cholera toxin B subunit (CTB, 1mg/ml in neutral phosphate buffer; Molecular Probes, Invitrogen Inc.) was unilaterally pressure-injected using a Hamilton syringe in dorsal or ventral distal forelimb muscles (dorsal: extensor carpi radialis longus and brevis, extensor digitorium communis, extensor digiti quarti and quinti; ventral: flexor carpi ulnaris, palmaris longus, flexor digitorium profundus radial and superficial head, flexor carpi radialis). 3 days post injection animals were transcardially perfused with 1% phosphate buffered saline (PBS) followed by 4% paraformaledhyde (PFA) in PBS, post-fixation for 4 h in 4% PFA, cryoprotection in 30% sucrose and sectioning in a series of four with a sliding microtome (SM 2000R, Leica). The spinal cervical vertebra C2 was used as a reference point.

#### Reconstruction of spinal motor pool representation

Two of the 4 consecutive series of coronal 40 μm spinal cord sections were used for quantification and reconstruction of labeled forelimb motor pools. Labeled motor neurons were captured on a fluorescent microscope (Axiovert 200, AxioCam HR, Axiovision software, Zeiss). Serial section images were aligned by means of 4 optical reference points and individual motoneurons were traced using the Reconstruct software [23].

#### Calculation of a scatter index for spreading of motor pools

For each animal the physiologically increased diameter of spinal sections at the level of the cervical enlargement was individually corrected for by post-section alignment (S1 Fig). A reference curve was defined for all sections using five points on the lateral edge of the spinal cord grey matter. For each individual section the intercept point (blue point in S1A Fig) between the reference curve and the perpendicular (blue line) to the main (mid-sagittal) symmetry axis through the central canal (white line) was calculated. Each section was then separately readjusted after spline interpolation of these intersection points.

In order to quantify the spreading of medial and lateral motor pools, we defined a scatter index (SI) by determining the area of an ellipse fitted to the samples. This is equivalent to computing the covariance matrix of the X- and Y-coordinates of the motor neuron traces for both motor pools, and multiplying its two eigenvalues: its eigenvectors are perpendicular and represent the major and minor axes of an ellipse centered at the data mean, with width adjusted to the directional standard deviations. The area of the ellipse is the product of the two eigenvalues and π, which defines the scatter index $\left[SI=\pi \cdot \prod_{i=1,2}eig_{i}\left(\text{cov}\left[x;y\right]\right)\right]$ for each motor pool.

Subsequently, the scattering of each motor pool in medio-lateral or dorso-ventral direction was determined by the calculation of the standard deviation of X- (medio-lateral) and Y-values (dorso-ventral). All results were calculated as mean values ± SEM using Prism 5.0 software (GraphPad Software, La Jolla). Unpaired 2-tailed Student’s t test was used for single comparisons.

### Electromyography

Experiments were carried out aseptically under anesthesia using Ketamine (0.1 mg/g) and Xylazine (0.01 mg/g). Mice were analyzed at 12 weeks of age and euthanized without recovering consciousness. Bipolar EMGs were fabricated from teflon-insulated multi-stranded fine wire (Science Products, Hofheim) as described [24]. A small incision was made above the brachial plexus to gain access to the forelimb nerves and muscles. Electrodes were inserted unilaterally into the *triceps brachii* (dorsal extensor) and antagonistic *biceps brachii* (ventral flexor). EMG activity was recorded in parallel from both muscles by stimulating the *musculocutaneous* nerve. Muscle activity was taped and analyzed using AxoScope software (Axon Instruments, Molecular devices).

### Immunohistochemistry

40 μm spinal cord sections were washed in PBS (3x 5 min) at room temperature and afterwards blocked in blocking solution for 30 min. Then, sections were incubated in blocking solution containing the primary antibodies or lectin in a humidified chamber for two nights at 4°C and washed in PBS containing 0.1% Triton X 100 (3x 5 min) and secondary antibody was applied in blocking solution over night at 4°C. The next day, sections were washed in PBS (3x 5 min) and mounted in Mowiol. The following antibodies and lectins were used for stainings: rabbit anti-vGAT (1:1000, Synaptic Systems), rabbit anti-vGlut1 (1:1000, Synaptic Systems), biotinylated Wisteria floribunda agglutinin (WFA) (1:200, Sigma-Aldrich (L1516). vGAT and vGlut1 stainings were carried out on consecutive sections.

### Analysis of neuromuscular junctions

Forelimb muscles were dissected from perfused, adult animals and fixed overnight in 4% PFA. After cryoprotection in 30% sucrose, muscles were cut at 80 μm using a cryostat and stained free floating. After washing in PBS (3x 15 min) the tissue was permeabilized in PBS containing 2% Triton X 100 for 30 min and blocked in blocking solution (4% BSA and 1% Triton X 100 in PBS) for another 30 min. Then sections were incubated with the primary antibodies (mouse anti-synaptophysin (Sigma, 1:100) and mouse anti-neurofilament 2H3 (DSHB, 1:50) overnight at 4°C. The next day, sections were washed (4 x 15 min) in PBS and rhodamin-labeled α-bungarotoxin (Invitrogen, 1:500) was applied in blocking buffer for 30 min. Afterwards, sections were incubated with the secondary antibody in blocking buffer (donkey anti-mouse Ax488, Invitrogen, 1:250) for 2 h. After the final washing steps (3 x 15 min in PBS) sections were mounted in Mowiol and analyzed using a confocal microscope. For the analysis the surface areas of at least 100 neuromuscular junctions were measured for each animal.

### Determination of synaptic input

At 12 weeks of age, ventral muscles of the lower forelimb (flexor carpi ulnaris, palmaris longus, flexor digitorium profundus radial and superficial head, flexor carpi radialis) were retrogradely labeled with CTB-Ax555 as described above. Consecutive spinal cord sections were stained for vGAT and vGlut1 and motor neurons were captured using a confocal laser scanning microscope (LSM510 Meta, Zeiss). At the central level of each motor neuron the number of labeled synapses was measured using ImageJ software.

## Results

### Impairments in motor coordination can be corrected by housing in enriched environment during a critical period after birth

**Fig 1:**
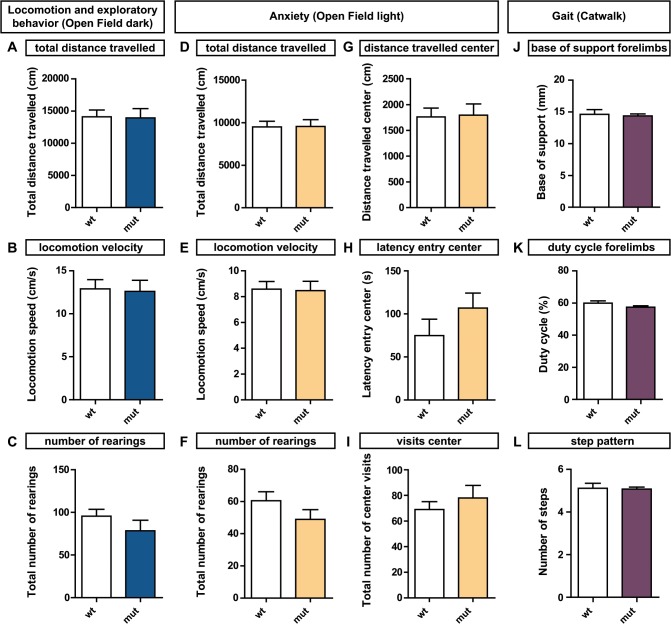
*Sema3F* mutants show normal behavior in the dark and light open field test. (A-C) Gross locomotion and exploratory behavior of *Sema3F* animals is analyzed at the age of 4 weeks in the dark open field. No significant differences are evident in (A) the total distance travelled (14100 ± 1069 cm vs. 13920 ± 1457 cm, p = 0.48), (B) the locomotion velocity (12.90 ± 1.07 cm/s vs. 12.61 ± 1.29 cm/s, p = 0.65) or (C) the number of rearings (95.60 ± 7.99 vs. 78.40 ± 12.40, p = 0.35). Statistical analysis: N = 10 for each group, Mann-Whitney test. * p < 0.05, ** p < 0.005, *** p < 0.001. (D-I) Anxiety related behavior is investigated in the light open field at 4 weeks of age. Overall locomotion or exploratory behavior is not affected in *Sema3F* mutants in the light open field as determined by (D) the total distance travelled (9505 ± 658.7 cm, N = 21 vs. 9543 ± 809.3 cm, N = 26, p = 0.86), (E) the locomotion velocity (8.586 ± 0.595 cm/s, N = 21 vs. 8.465 ± 0.725, N = 26, p = 0.72) or (F) the number of rearings (60.52 ± 5.57, N = 21, vs. 48.89 ± 6.06, N = 19, p = 0.17). The determination of (G) the distance travelled in the center (1759 ± 172.1 cm, N = 21 vs. 1796 ± 215.8 cm, N = 26, p = 0.66), (H) the time until the first center entry (74.86 ± 19.07 s, N = 21 vs. 106.8 ± 17.59 s, N = 26, p = 0.10) and (I) the number of center visits (69.10 ± 6.09, N = 21 vs. 78.00 ± 9.91, N = 26, p = 0.47) does not reveal any anxiety related behavior in *Sema3F* mutants. (J-L) Gait of *Sema3F* animals was analyzed 9 weeks after birth using the CatWalk analysis system. No significant differences were found in (J) the forelimb base of support (14.64 ± 0.73 mm, N = 9 vs. 14.37 ± 0.32 mm, N = 9; p = 1.0), (K) the duty cycle of the forelimbs (59.88 ± 1.43%, N = 9 vs. 57.42 ± 0.86%, N = 9; p = 0.11) or (L) the step pattern of the animals (5.11 ± 0.23, N = 9 vs. 5.07 ± 0.09, N = 9; p = 0.58).Statistical analysis: Mann-Whitney test. * p < 0.05, ** p < 0.005, *** p < 0.001.

**Fig 2:**
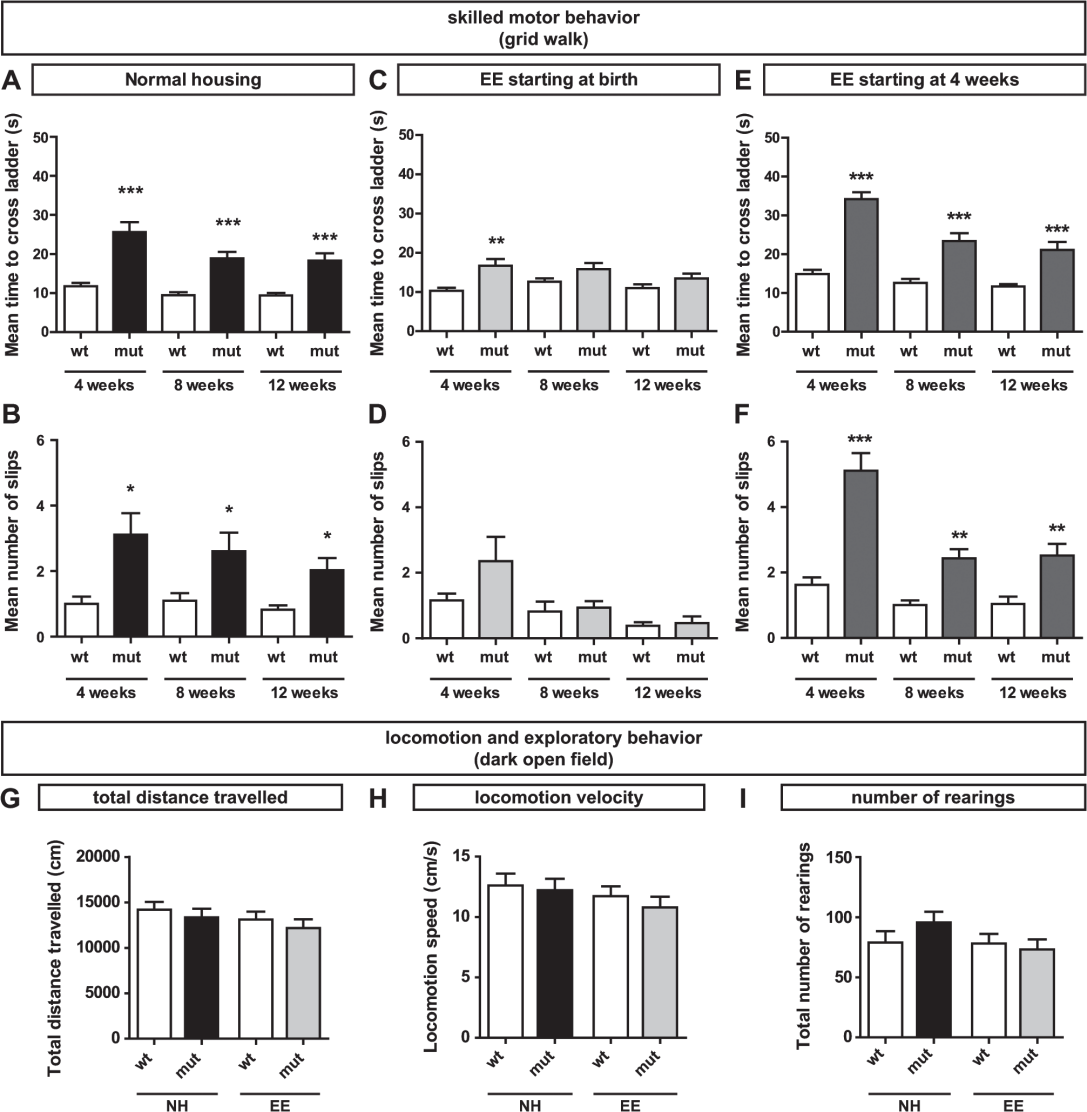
Motor coordination of *Sema3F* mice after housing in different environmental conditions. (A-F) Motor coordination deficits were analyzed using the ladder rung test. (A) Under normal housing conditions, *Sema3F* mutants need significantly more time to cross the ladder with irregularly spaced bars than littermate controls at each time point tested (4 weeks: 11.74 ± 0.86 s, N = 14 vs. 25.58 ± 2.56 s, N = 12, p < 0.001; 8 weeks: 9.48 ± 0.75 s, N = 14 vs. 18.92 ± 1.64 s, N = 12, p < 0.001; 12 weeks: 9.41 ± 0.67 s, N = 13 vs. 18.31 ± 1.89 s, N = 12, p < 0.001, Improvement mut 4–12 weeks: p = 0.03). (B) *Sema3F* mutants also show a significantly increased number of slips (4 weeks: 1.00 ± 0.22, N = 14 vs. 3.11 ± 0.66, N = 12, p < 0.05; 8 weeks: 1.10 ± 0.23, N = 14 vs. 2.61 ± 0.56, N = 12, p < 0.05; 12 weeks: 0.82 ± 0.14, N = 13 vs. 2.03 ± 0.37, N = 12, p < 0.05). (C) After enriched environment housing starting at birth the motor performance of *Sema3F* mutants reaches wildtype levels at 8 weeks after birth (4 weeks: 10.37 ± 0.76 s, N = 10 vs. 16.74 ± 1.71, N = 9, p < 0.005; 8 weeks: 12.63 ± 0.90 s, N = 10 vs. 15.85 ± 1.54 s, N = 9, p = 0.14; 12 weeks: 11.00 ± 0.98 s, N = 10 vs. 13.48 ± 1.19 s, N = 9, p = 0.07). (D) These animals never show a significant difference in the number of slips compared to wildtype littermates (4 weeks: 1.00 ± 0.21, N = 10 vs. 1.67 ± 0.49, N = 9, p = 0.36; 8 weeks: 0.83 ± 0.16, N = 10 vs. 1.07 ± 0.17, N = 9, p = 0.31; 12 weeks: 0.47 ± 0.09, N = 10 vs. 0.59 ± 0.15, N = 9, p = 0.50). (E) Enriched environment housing starting at 4 weeks after birth does not improve the motor performance of *Sema3F* mutants. (4 weeks: 15.19 ± 1.17 s, N = 19 vs. 34.06 ± 2.09 s, N = 11,p < 0.001; 8 weeks: 12.61 ± 1.01 s, N = 19 vs. 23.91 ± 2.17 s, N = 11, p < 0.001; 12 weeks: 11.75 ± 0.62 s, N = 19 vs. 21.55 ± 2.22 s, N = 11, p < 0.001). (F) Also the number of slips from the ladder is significantly increased at each time point after enriched environment housing starting at 4 weeks (4 weeks: 1.81 ± 0.24, N = 19 vs. 5.30 ± 0.59, N = 11, p < 0.001; 8 weeks: 1.00 ± 0.15, N = 19 vs. 2.24 ± 0.32, N = 11, p < 0.005; 12 weeks: 1.00 ± 0.24, N = 19 vs. 2.394 ± 0.3140, N = 11, p < 0.005). Statistical analysis: Mann-Whitney test. * p < 0.05, ** p < 0.005, *** p < 0.001. (G-I) Improved performance in the grid walk test is not caused by alterations in general locomotion or exploratory behavior. At the age of 12 weeks, animals that were housed in normal or enriched housing conditions starting at birth do not reveal any significant differences in the open field test as determined by (G) the total distance that was traveled (NH: 14190 ± 867.2 cm, N = 16 vs. 13350 ± 951.9 cm, N = 20; EE: 13110 ± 871.3 cm, N = 23 vs. 12160 ± 983.2 cm, N = 20; ANOVA: p = 0.53), (H) the locomotion speed (NH: 12.62 ± 0.96 cm/s, N = 16 vs. 12.22 ± 0.95 cm/s, N = 20; EE: 11.73 ± 0.81 cm/s, N = 23 vs. 10.79 ± 0.89 cm/s, N = 20; ANOVA: p = 0.54) and the total number of rearings (NH: 79.00 ± 9.41, N = 16 vs. 95.85 ± 8.82, N = 20; EE: 78.17 ± 8.16, N = 23 vs. 73.25 ± 8.28, N = 20; ANOVA: p = 0.27). Statistical analysis: One-way ANOVA. * p < 0.05, ** p < 0.005, *** p < 0.001.

Loss of *Sema3F* signaling causes severe axon wiring defects of ventrally projecting motor axons in the brachial spinal cord [5]. During embryonic development, these deficits were investigated thoroughly, however, the resulting effects on postnatal animals did not receive the same attention. To address this issue and since motor axons are affected during development, we performed behavioral tests focusing on different aspects of locomotion in these animals. We found no effects on general locomotor activity or anxiety related behavior tested in the open field or on gait using the CatWalk analysis system (Fig 1). Thus, we next aimed to investigate more specific skilled motor functions and tested forelimb-hindlimb coordination on a horizontal ladder with irregular bars. At 4 weeks of age, *Sema3F* mutants needed significantly more time to cross the ladder than their wildtype littermates (Fig 2A). Furthermore, mutant animals revealed deficits in fine motor control since they showed a significantly increased number of slips during this task (Fig 2B). Over the time course of 8 weeks these animals were able to improve their performance on the ladder, however, they never reach wildtype levels.

The improvement in motor performance from 4 to 12 weeks of age indicates adaptive changes during postnatal development. Since housing mice in enriched environments has been shown to have positive effects on motor behavior after spinal cord injury and in disease [16, 25], we tested the effects of enriched environment housing on the motor performance of *Sema3F* mutants. Animals were housed in enriched environment cages that were considerably larger and contained material to stimulate motor behavior such as a running wheel, nesting material, and a small horizontal ladder with regular bars. Already at 4 weeks of age, the effect of enriched environment housing on the motor performance of the animals becomes evident. Since *Sema3F* wildtypes already show a very high level of motor coordination in normal housing conditions, enriched environment does not affect their performance in the test. In contrast, *Sema3F* mutants that were housed under enriched environment conditions starting at birth, show an improved performance compared to mutants in normal housing conditions (S2 Fig and Fig 2C and 2D). Nevertheless, at this time point *Sema3F* mutants still performed significantly worse than their wildtype littermates (Fig 2C and 2D). At the age of 8 weeks, however, the motor deficits were compensated and no significant difference between the performance of wildtype and mutant animals was detectable. In order to test if an increased locomotion or exploratory behavior might be responsible for this effect we tested mice in an open field at the age of 12 weeks and found no difference between animals that were housed normally or in an enriched environment starting at birth (Fig 2G–2I).These data suggest that enriched environment housing induces plastic changes that allow for the compensation of deficits in motor coordination in *Sema3F* mutants.

For many different systems it has already been shown that the rearrangement of neuronal circuits is restricted to specific time windows during development that are called critical periods [6]. To investigate whether the compensatory effects in *Sema3F* mice are also limited to a specific developmental period, we have analyzed the effects of a delayed exposure to enriched environment housing: After having spent the first 4 weeks after birth in normal housing conditions the animals were then changed to enriched housing conditions. Indeed, these animals showed a similar performance as animals housed in normal conditions. *Sema3F* mutants showed an improvement of their performance over the time course of 8 weeks, but at each time point tested (4, 8 and 12 weeks after birth) mutant animals performed significantly worse than their wildtype littermates (Fig 2E and 2F).

Together, these data suggest that *Sema3F* mutant mice are able to improve their motor performance due to experience-induced changes that take place within a critical period in the first 4 weeks after birth.

### *Sema3F* mutants reveal misinnervation of the dorsal forelimb musculature in all housing conditions

**Fig 3:**
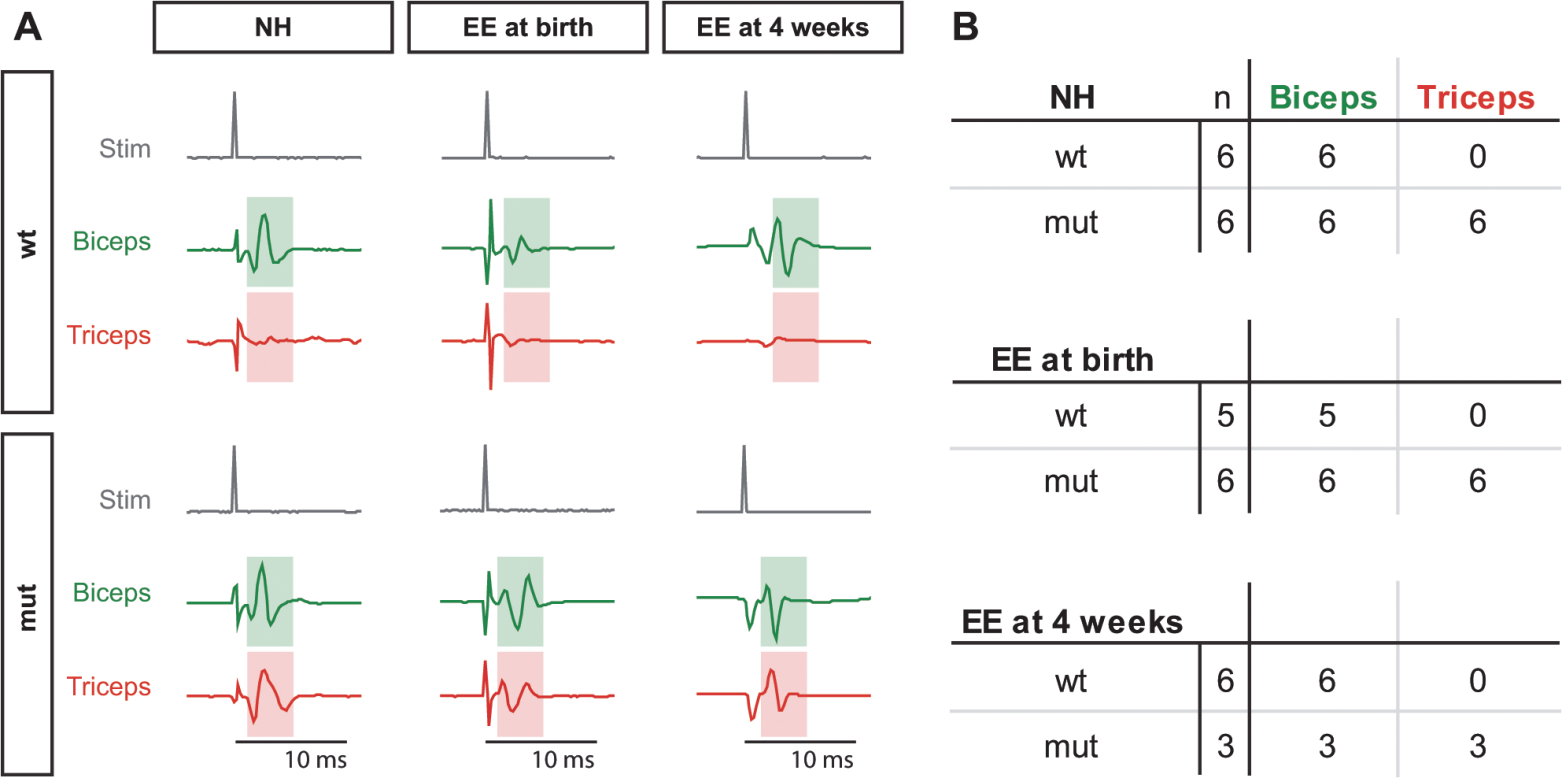
Electromyography reveals innervation defects in *Sema3F* mutants under all housing conditions. (A) After stimulation of the *musculocutaneous* nerve, wildtype animals show a signal in the *biceps brachii* (green box) while the *triceps brachii* is not activated (red box) and only the stimulation artefact is visible. In *Sema3F* mutants the stimulation of the *musculocutaneous* nerve leads to the activation of *biceps brachii* and *triceps brachii* muscles at the same time. This was observed in all housing conditions (normal housing, enriched environment starting at birth and enriched environment starting at 4 weeks). (B) Quantification of activation signals. The table displays the total number of tested animals and the number of animals showing a signal in the respective muscle after activation of the *musculocutaneous* nerve.

*Sema3F* mutants show axon guidance errors during embryonic development and postnatal coordination deficits that were compensated by housing in an enriched environment starting at birth. In order to rule out that the observed differences in motor coordination are evoked by alterations in forelimb innervation that could have taken place in the late phase of embryonic development, we analyzed the functionality of forelimb innervation by electromyography. Therefore, we stimulated the *musculocutaneous* nerve that is responsible for the innervation of ventral forelimb muscles, esp. the *biceps brachii*, and recorded signals from *biceps brachii* and *triceps brachii* muscles. In wildtype animals, we found signals exclusively in *biceps brachii*, however, *Sema3F* mutants showed signals in *biceps brachii* and *triceps brachii* musculature. This was the case for all housing conditions (normal housing (NH), enriched environment starting at birth (EE at birth) and enriched environment starting at 4 weeks (EE at 4 weeks)) (Fig 3).

Thus, our electrophysiological data match the described embryonic pathfinding deficits, showing that in *Sema3F* mutants the ventrally projecting *musculocutaneous* nerve is also innervating muscles in the dorsal side of the limb. Furthermore, the observed improvement in motor coordination of *Sema3F* mutants after enriched environment housing starting at birth was not accompanied by changes in forelimb innervation. Since an additional investigation of neuromuscular junctions in the forelimb musculature of adult *Sema3F* animals revealed comparable surface areas after normal or enriched environment housing starting at birth (S3 Fig), we did not find any adaptions in the peripheral nervous system of these animals, supporting the hypothesis that plastic rearrangements in the central nervous system are responsible for the described effects.

### Neuroanatomical rearrangements corroborate a critical period for adaptive plasticity in the spinal cord of *Sema3F* mutants

**Fig 4:**
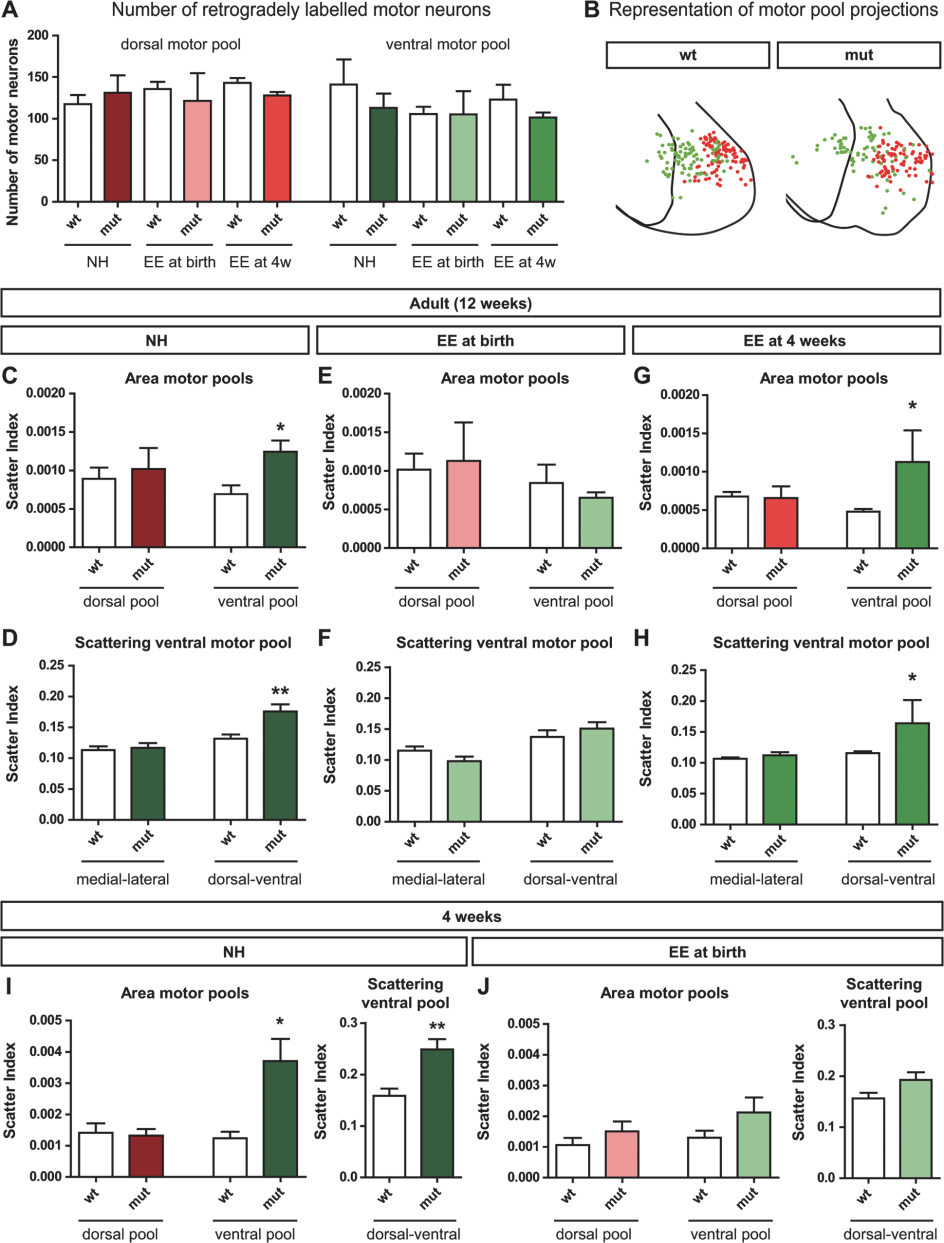
Enriched environment housing starting at birth induces neuroanatomical rearrangements of spinal motor pools. At 12 weeks of age, motor neurons were retrogradely labeled by injection of Alexa Fluor-conjugated CTBs into the dorsal (red) or ventral (green) muscles of the distal forelimb. (A) The number of retrogradely traced motor neurons in the respective motor pool was comparable in wildtypes and mutants of all housing conditions (dorsal pool: p = 0.83; ventral pool: p = 0.68; N ≥ 3 for each group, one-way ANOVA). (B) Motor pools were reconstructed from labeled motor neurons of the brachial spinal cord. The schematics show the projection of all motor neurons along the anterior-posterior axis. The first and last outline of the ventral horn grey matter are indicated (C) In normal housing conditions the medial motor pool of adult animals is significantly larger in *Sema3F* mutants compared to their wildtype littermates. In contrast, the lateral motor pool remains unchanged (ventral: 0.00069 ± 0.00012, N = 7 vs. 0.00124 ± 0.00015, N = 5; p < 0.05; dorsal: 0.00089 ± 0.00015, N = 7 vs. 0.00102 ± 0.00027, N = 5; p = 0.66; Student’s t-test). (D) A specific scattering of the pool is evident in the dorsal-ventral direction, while the medial-lateral dimensions of the pool are not affected (dorsal-ventral: 0.13 ± 0.0076, N = 7 vs. 0.18 ± 0.0114, N = 5, p < 0.01; medial-lateral: 0.11 ± 0.0061, N = 7 vs. 0.12 ± 0.0080, N = 5, p = 0.73, Student’s t-test). (E + F) After housing in an enriched environment starting at birth, plastic rearrangements become evident and no motor pool shows a significantly altered area between wildtype and mutant animals (area dorsal: 0.00102 ± 0.00021, N = 5 vs. 0.00113 ± 0.00050, N = 3, p = 0.81; area ventral: 0.00084 ± 0.00024, N = 5 vs. 0.00065 ± 0.00007, N = 3, p = 0.57; scattering medial-lateral: 0.1149 ± 0.0068, N = 5 vs. 0.0979 ± 0.0074, N = 3, p = 0.16; scattering dorsal-ventral: 0.1372 ± 0.0108, N = 5 vs. 0.1506 ± 0.0104, N = 3, p = 0.44, Student’s t-test). (G) Enriched environment starting at 4 weeks does not induce these changes. Here, only the lateral motor pool appears normal while the medial motor pool is still significantly larger in mutants compared to wildtype littermates (dorsal: 0.00065 ± 0.00006, N = 8 vs. 0.00066 ± 0.00015, N = 3, p = 0.94; ventral: 0.00045 ± 0.00004, N = 8 vs. 0.00113 ± 0.00041, N = 3, p < 0.05, Student’s t-test). (H) The analysis of the specific scattering reveals an extension of the pool in dorsal-ventral direction (medial-lateral: 0.1068 ± 0.0021, N = 8 vs. 0.1121 ± 0.004982, N = 3, p = 0.26; dorsal-ventral: 0.1157 ± 0.0031, N = 8 vs. 0.1642 ± 0.0375, N = 3, p < 0.05, Student’s t-test). (I and J) Already at 4 weeks after birth the plastic rearrangements of the medial motor pool due to enriched environment housing are evident. While the medial pool shows a significant dorsal-ventral scattering in normally housed animals (I) (area dorsal: 0.00141 ± 0.00030, N = 5 vs. 0.00132 ± 0.00021, N = 5, p = 0.81; area ventral: 0.00124 ± 0.00020, N = 5 vs. 0.00371 ± 0.00071, N = 5, p < 0.05; scattering dorsal-ventral: 0.1587 ± 0.0139, N = 5 vs. 0.2486 ± 0.0204, N = 5, p < 0.01, Student’s t-test), in animals that were housed in an enriched environment starting at birth the pool has normal dimension (J) (area dorsal: 0.00106 ± 0.00023, N = 5 vs. 0.00151 ± 0.00032, N = 5, p = 0.29; area ventral: 0.00130 ± 0.00023, N = 5 vs. 0.00212 ± 0.00049, N = 5, p = 0.17; scattering dorsal-ventral: 0.1564 ± 0.0109, N = 5 vs. 0.1923 ± 0.0154, N = 5, p = 0.09, Student’s t-test). * p < 0.05, ** p < 0.005, *** p < 0.001.

Since behavioral tests and electromyography data suggest adaptive plasticity in the central nervous system of *Sema3F* mutants we next investigated the phenotype at a neuroanatomical level. Therefore, we injected Alexa Fluor-conjugated CTBs into the dorsal and ventral musculature of the lower forelimb for retrograde visualization of the innervating motor pools in the spinal cord. Quantification of the number of retrogradely traced motor neurons revealed no significant differences between wildtypes and mutants or between animals of the three different housing conditions (Fig 4A).

To analyze the distribution of these motor neurons in more detail we reconstructed the localization the entire pools and we found that motor neurons innervating the ventral side of the limb appeared to be less organized in *Sema3F* mutants and spread into the dorsally projecting motor pool (Fig 4B). To quantify this finding we calculated the scatter index, which characterizes the spreading of each motor pool within the spinal cord. Interestingly, in normally housed *Sema3F* mutants ventrally projecting LMCm neurons occupied a larger area than in their wildtype littermates, while the lateral pool showed no significant alterations. This is very reminiscent of the defects observed during embryonic development, where only the LMCm neurons project their axons aberrantly [5]. A closer investigation of this area revealed that the spreading of the pool is specifically scattered in the dorsal-ventral axis, while the medial-lateral scattering of the pool appeared to be not affected (Fig 4C and 4D). A similar effect was found for animals that were housed in an enriched environment starting at 4 weeks. The medial pool shows a significantly increased area with a specific scattering in the dorsal-ventral direction (Fig 4G and 4H). In contrast, this increased scattering of ventrally projecting neurons was not found in *Sema3F* mutants that were housed in an enriched environment starting at birth when they were compared to littermate controls (Fig 4E and 4F).

In order to investigate to which extent the organization of the medial motor pool is already altered within the first four postnatal weeks, we performed the neuroanatomical analysis also in young animals at this time point. Our data show that already at four weeks *Sema3F* mutants that were housed in an enriched environment do not reveal any significant alterations in the dimensions of their ventrally projecting motor pool, while normally housed animals show the significant spreading of the pool in a dorsal-ventral direction (Fig 4I and 4J), suggesting that at this time the plastic rearrangements within the spinal cord are already completed.

Thus, our neuroanatomical data correlate nicely with the findings from behavioral analyses (S4 Fig) and support the hypothesis that enriched environment housing induces plastic rearrangements in the spinal cord within a critical period in the first four postnatal weeks after birth.

### Perineuronal nets play no major role in the regulation of plasticity in *Sema3F* mutants

**Fig 5:**
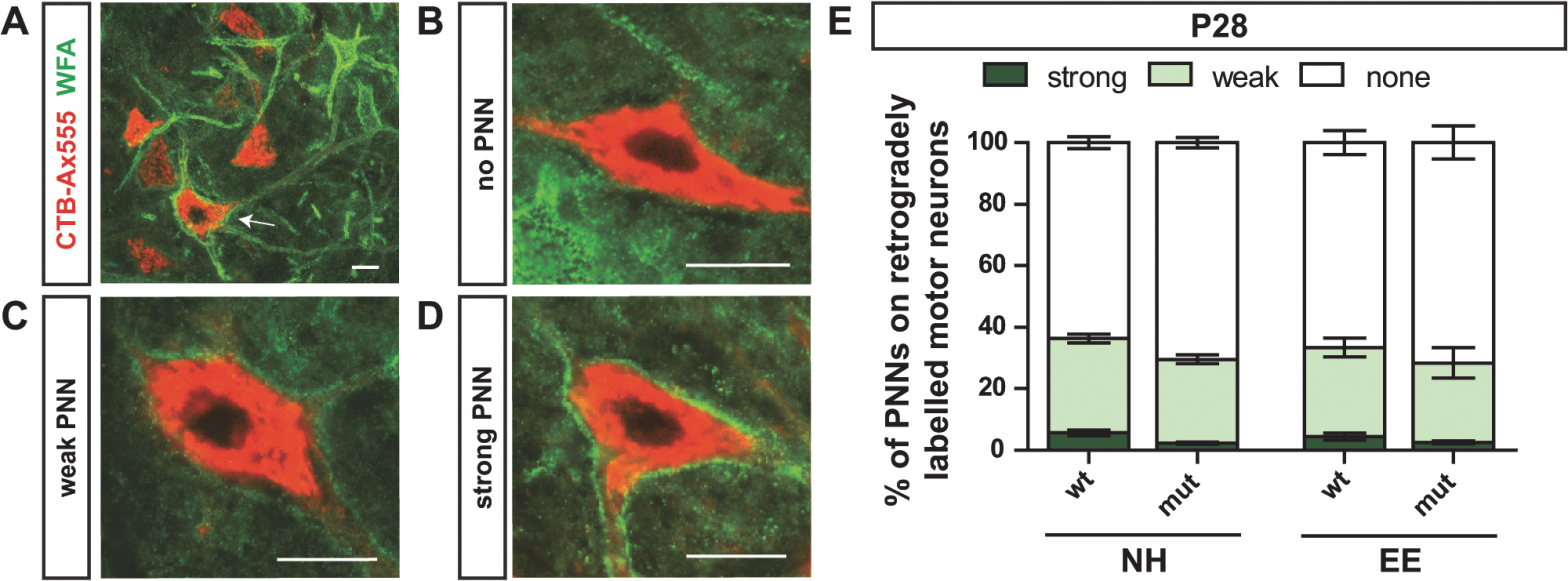
Most spinal motor neurons are not protected by PNNs. (A) In the adult spinal cord only few motor neurons retrogradely labeled from the distal ventral forelimb show PNNs (arrow). (B—D) Examples of motor neurons with no, weak, or strong PNNs, respectively. (E) At 4 weeks of age, when the critical period for adaptive plasticity is closed, more than 65% of traced motor neurons are not covered by PNNs, regardless of the housing conditions (NH: wt: 63.6 ± 3.4%; mut: 70.4 ± 3.0%; EE: wt: 66.6 ± 6.8%, mut: 71.6 ± 9.4%; p = 0.43). Weak PNNs are found on less than 30% (NH: wt: 30.7 ± 2.5%; mut: 27.2 ± 2.5%; EE: wt: 29.0 ± 5.3%, mut: 25.9 ± 8.6%; p = 0.72) and less than 5% of motor neurons show strong PNNs (NH: wt: 5.7 ± 1.7%; mut: 2.4 ± 0.6%; EE: wt: 4.4 ± 2.0%, mut: 2.6 ± 0.8%; p = 0.06). Statistical analysis: N = 3 for each group, one-way ANOVA. * p < 0.05, ** p < 0.005, *** p < 0.001. Scale bar: 20 μm.

The closure of critical periods for plasticity in the nervous system has been closely related to the development of perineuronal nets (PNNs) for several areas of the nervous system [26–28]. These structures of extracellular matrix form around cell bodies as a tight meshwork and constitute a physical barrier for plastic changes by preventing the formation of new synapses [20, 29]. In several cases it has been shown that enriched environment housing can influence the formation of PNNs and thereby extend the period for adaptive plasticity [26, 28]. Therefore, we investigated whether PNNs play a role for the adaptive plasticity induced by environmental enrichment in the spinal cord of *Sema3F* mutants. Upon staining with *wisteria floribunda* agglutinin (WFA), we found that the formation of PNNs around motor neurons in the spinal cord is very heterogeneous (Fig 5A) and most motor neurons are not protected by PNNs. At 4 weeks, when the critical period is closed, only about 35% of ventrally projecting motor neurons that were labeled by retrograde tracing have PNNs and in less than 5% they form a very tight meshwork (Fig 5B–5E). Furthermore, no significant difference in the localization and density of PNNs was found between the different housing groups and also during their formation between postnatal day 7 and 21 no significant changes became evident (S5 Fig). This result suggests that the formation of PNNs plays no important, if any, role in the regulation of adaptive plasticity in the spinal motor system of *Sema3F* mice.

### Enriched environment housing alters the balance of excitatory-inhibitory input on motor neurons

**Fig 6:**
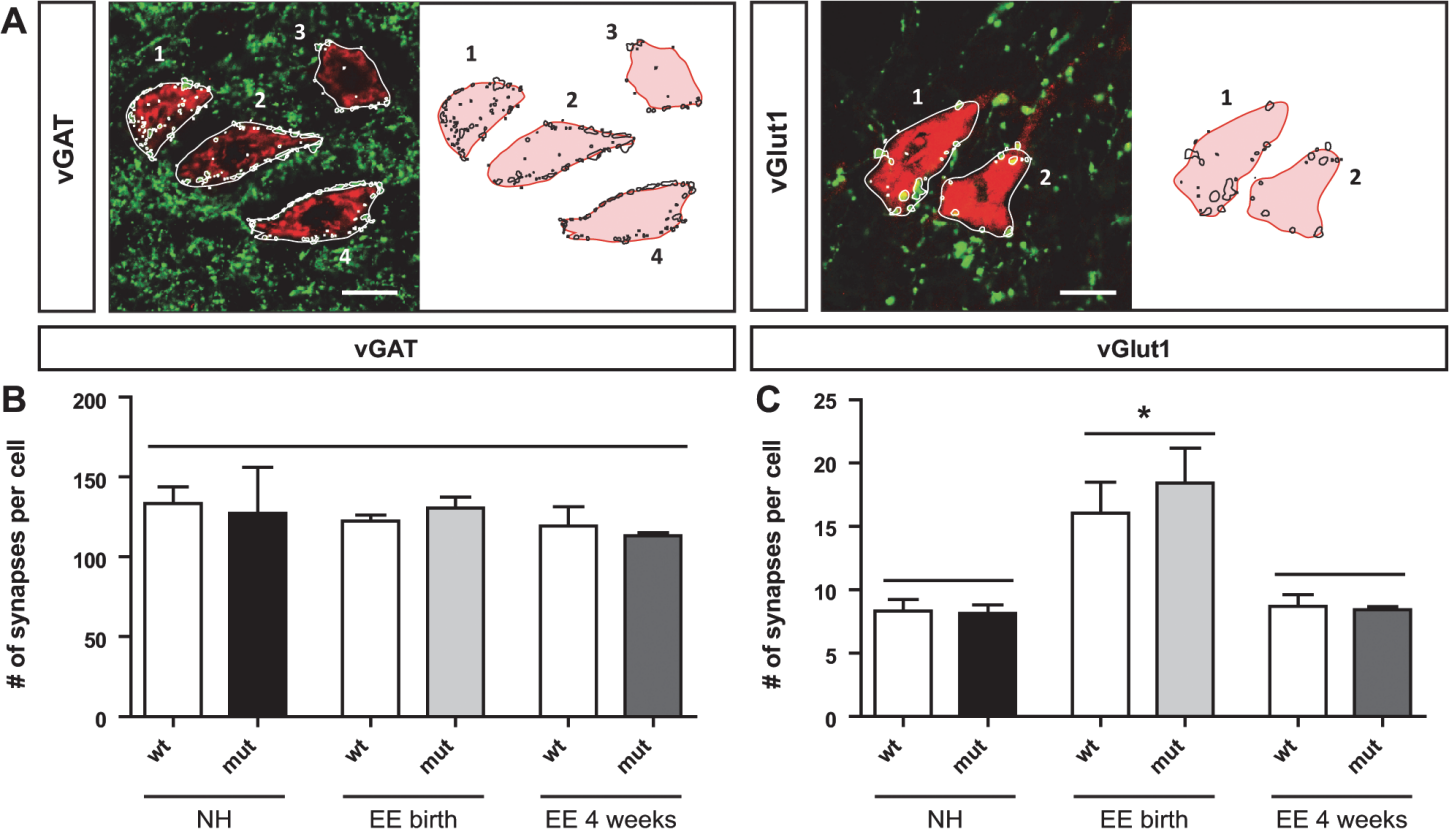
Excitatory-inhibitory balance of synaptic input is shifted by enriched environment housing. (A) Example of excitatory (vGlut1) and inhibitory (vGAT) synapses on retrogradely labeled motor neurons of 12 week old animals. (B) The number of inhibitory synapses on traced motor neurons remains unchanged between wildtypes and mutants of all housing conditions (normal housing (wt: 133.5 ± 10.52, mut: 127.3 ± 28.76), enriched environment starting at birth (wt: 122.5 ± 3.85, mut: 125.0 ± 6.63), and enriched environment starting at 4 weeks (wt: 119.4 ± 12.0, mut: 113.2 ± 2.15); N = 3 for each group, p = 0.91, one-way ANOVA). (C) Between *Sema3F* wildtypes and mutants, the number of excitatory synapses is not significantly altered (NH: wt: 8.32 ± 0.90, mut: 8.13 ± 0.67, p = 0.87; EEbirth: wt: 15.99 ± 2.51, mut: 16.81 ± 3.88, p = 0.75; EE4: wt: 8.70 ± 0.93, mut: 8.43 ± 0.24; N = 3 for each group, Students t-test), however, after enriched environment housing starting at birth the number of excitatory synapses were significantly increased when compared to normal housing conditions or enriched environment starting at 4 weeks (NH vs. EEbirth: wt: p = 0.040, mut: p = 0.022; EEbirth vs. EE4: wt: p = 0.047, mut: p = 0.023, N = 3 for each group, Students t-test). Statistical analysis: N = 3 for each group, Students t-test. * p < 0.05, ** p < 0.005, *** p < 0.001. Scale bar: 20 μm.

Since activity shapes the neuronal circuits during postnatal development and plasticity in the central nervous system has been shown to be affected by the balance of excitatory and inhibitory synaptic inputs [26], we next analyzed the synaptic input on motor neurons innervating the lower ventral forelimb by staining against the vesicular transporters vGlut1 and vGAT for excitatory and inhibitory synapses, respectively. For the analysis of the synapses on each motor neuron the number of stained particles on the cell body was counted using the ImageJ software (Fig 6A). We found that absence of Sema3F does not affect the formation of excitatory or inhibitory synapses per se: the number of synapses remained unchanged between mutants and wildtypes, regardless of their housing conditions (Fig 6B and 6C). However, we observed that the housing conditions had a significant impact on the balance of excitatory and inhibitory synapses: while the number of inhibitory synapses remained unchanged between all groups (Fig 6B), a significant increase in the number of excitatory synapses on ventrally projecting motor neurons after enriched environment housing starting at birth was detected when compared to normally housed animals. This effect was not evident after enriched environment housing starting at 4 weeks (Fig 6C). Thus, within the first four postnatal weeks enriched environment housing induces a shift of the excitatory-inhibitory balance towards excitation.

## Discussion

Plasticity of neuronal circuits has been studied for decades, however, we are still far from a complete understanding of the mechanisms underlying adaptive changes in the nervous system. In the spinal cord, the analysis of plastic rearrangements has mainly focused on models of experimental spinal cord injury, which is accompanied by several disadvantages such as difficulties in reproducibility or injury related reactions within the spinal cord tissue. To address these challenges, we established a new non-injury based model for the investigation of plasticity in the spinal motor system. For this purpose, we used the genetically induced axon miswiring in *Sema3F* knockout mice as a model and investigated locomotor behavior and ability for compensational rearrangements during postnatal development.

### Axon miswiring in *Sema3F* mutants causes coordination deficits that are accompanied by structural rearrangements in the spinal motor pools

Lack of Sema3F signaling during embryonic development has severe consequences for the establishment of neural circuitry. Thus, on the spinal level, Neuropilin-2 expressing motor axons originating in the medial aspect of the lateral motor column (LMCm) are not repelled by the secreted chemorepulsive guidance cue Sema3F that is expressed in the dorsal limb mesenchyme, which causes guidance deficits and miswiring of these motor axons [5]. We showed that these wiring deficits are still persistent in adulthood, since electrophysiological stimulations of the *musculocutaneous* nerve revealed atypical signals in the *triceps brachii* muscle of *Sema3F* mutants (Fig 3). This suggests that the wiring of the misprojecting motor axons is not corrected during naturally occurring motor neuron death and axon pruning in the late embryonic and early postnatal phase [30, 31].

Furthermore, our data show that these defects in the spinal motor circuitry do not affect general locomotion and anxiety related behavior in *Sema3F* knockout mice, as identified by the Open Field and CatWalk test (Fig 1). Nevertheless, in more complex functions like forelimb-hindlimb coordination these mutants show a significantly weaker performance compared to their wildtype littermates (Fig 2A and 2B). We found that these behavioral deficits correlate with structural rearrangements within the spinal cord. In *Sema3F* mutants, ventrally projecting motor neurons are less densely packed within their pool compared to wildtype littermates, which results in a specific increase in the dorsal-ventral scattering of motor neuron positions (Fig 4C and 4D). Even though the exact mechanisms that govern the formation of muscle specific motor pools are not known yet, studies during embryonic development already suggest the involvement of secreted class 3 semaphorins in this process, due to the distinct expression patterns of the semaphorins and their receptors [32]. Thus, our results corroborate a putative function of Sema3F signaling in the formation and consolidation of muscle specific spinal motor pools.

### During a critical period, unspecific motor training by enriched environment housing induces adaptive plasticity with effects on motor coordination

Our experiments with animals raised in an enriched environment starting at birth revealed a significantly enhanced motor performance of *Sema3F* mutants, which is accompanied by a reorganization of the spinal motor pools already at 4 weeks of age (Figs 2 and 4), suggesting that these plastic changes are induced in the early postnatal stage. Furthermore, a lack of behavioral and neuroanatomical alterations in animals that were housed in an enriched environment from the age of 4 weeks implies a critical period for plasticity in the spinal motor system. This finding is supported by the existence of critical periods for adaptive changes in various other systems like the visual and auditory cortex or synapse elimination at neuromuscular junctions [6]. Additionally, the unchanged number of retrogradely labeled motor neurons in all environmental conditions (Fig 4A) excludes earlier (late embryonic or perinatal) effects like naturally occurring motor neuron death to be involved in the rearrangement of the motor pools [33, 34].

Thus, our data suggest a critical period within the first four postnatal weeks, in which enriched environment housing may induce plastic rearrangements in the spinal motor system. During the first two postnatal weeks an active use of toys or the running wheel is not possible, since newborn pups do not start to walk until postnatal day 12–21 [35]. However, also in the very early postnatal phase the enriched environment may play a very important role, as several studies have shown that enriched environment housing influences maternal care behavior including enhanced licking, grooming and physical contact that causes an increased tactile stimulation of newborn animals [14, 36]. Thus, already in neonatal pups activity-dependent mechanisms are induced by enriched environment housing that have been shown to induce plastic rearrangement of neuronal circuits in different model systems [12, 16, 25].

### Enriched environment influences the formation of new synapses but not the timing of the critical period for adaptive plasticity

To gain further insights in the mechanisms underlying adaptive plasticity in the spinal motor system we investigated the affected motor neurons in greater detail. Due to their reported relevance for the closure of critical periods [20, 27, 29] we analyzed the formation of perineuronal nets (PNNs) on motor neurons after normal and enriched environment housing. Surprisingly, we found no significant difference between the different housing groups. This is an interesting result since studies in most of the investigated model systems have revealed a positive [37] or negative [28, 38] effect of enriched environment housing on the formation of PNNs and the related critical period for plasticity. Furthermore, our results revealed that at 4 weeks of age most of the labeled spinal motor neurons (about 65%) were not protected by this specialized form of extracellular matrix and only about 5% that were covered by strong PNNs. This is corroborated by a study that examined the distribution of extracellular matrix components within the adult spinal cord of rats and found that only about 30% of the motor neurons in the ventral horn exhibit PNNs [39]. Thus, even though PNNs are formed in the spinal cord, they seem to play only a minor role in the plasticity related development of motor neurons, since only a small proportion of motor neurons exhibit PNNs in the spinal motor system and the formation of PNNs is not affected by activity-dependent mechanisms due to enriched environment housing.

Next, we analyzed the formation of new synapses on ventrally projecting motor neurons (Fig 6), since the balance of excitatory and inhibitory input has been shown to influence plasticity in the nervous system [26, 40]. We found that enriched environment housing does not affect the formation of inhibitory synapses on ventrally projecting motor neurons. Since the control of inhibitory synaptic input was shown to influence critical period regulation [26, 41], this result is corroborated by the findings of our PNN analysis, indicating that enriched environment housing has no influence on the timing of the critical period in the spinal motor system. Furthermore, our analysis showed an increase in the number of excitatory synapses on LMCm neurons. This is in agreement with various studies revealing that synaptogenesis is induced by enriched environment in models of normal and disease related neuronal circuits [25, 42, 43]. Furthermore, also strength training but not skill training was shown to induce the formation of additional excitatory synapses on spinal motor neurons [44]. Interestingly, in those strength trained animals the cortical motor map was not reorganized even though motor performance was improved indicating that plasticity in the spinal cord is responsible for the observed effects [45]. However, the organization of the spinal motor pools was not investigated in these studies. Thus, our results give evidence for plasticity in the spinal motor system by the formation of additional excitatory synapses on ventrally projecting motor neurons.

### Relevance of plasticity within the sensory-motor cortex of *Sema3F* mutants

We showed that structural rearrangements in spinal motor pools correlate with behavioral improvement after enriched environment housing starting at birth. However, also in normally housed animals a significant improvement of locomotor performance becomes evident between 4 and 12 weeks of age. This suggests that compensational mechanisms or motor learning might help to cope with the described impairments [46, 47]. To exclude that the repetitive testing of the animals affected the results we tested animal cohorts at single time points only (8 and 12 weeks) and found no significant difference between the age-matched groups (data not shown). Thus, task specific motor learning is not involved in the amelioration of their motor deficits and suggests that other compensational mechanisms are activated in the nervous system of *Sema3F* mutants. Furthermore, we found that structural alterations of the spinal motor pool areas with a specific dorsal-ventral spreading are equally evident at 4 and 12 weeks, implying that plastic rearrangements within the spinal cord are not responsible for the behavioral improvement.

Sema3F is known to be expressed in several different brain regions [19, 48, 49], however an involvement of Sema3F in the formation of circuitries that might contribute to motor coordination has not been shown yet. Therefore, it would be interesting to see if alterations in the motor cortex might account for the improved motor behavior. Unfortunately, a muscle specific trans-synaptic retrograde labeling of motor areas using a genetically modified rabies virus is currently technically not feasible in adult animals thereby preventing us from analyzing the cortical motor areas that might contribute to the observed adaptive changes. However, since motor learning is not restricted to a critical period during early postnatal development [50], it is likely that plasticity in the sensory-motor cortex might enable these animals to adapt to their neuroanatomical deficits and allows them to improve their performance to a certain extent.

In conclusion, our data show that within the first four postnatal weeks, enriched environment housing is able to induce adaptive structural plasticity in the spinal motor system of *Sema3F* mutant animals that lead to neuroanatomical rearrangements and improvement of motor coordination. After this critical period no adaptive changes by enriched environment housing were possible anymore. Furthermore, we showed that plastic rearrangements go along with a shift in the balance of excitatory-inhibitory input on the affected motor neuron pool and that the formation of PNNs does not play an important role during this process. Thus, by taking advantage of the genetically induced axonal miswiring in *Sema3F* knockout mice, we established a new non-injury based model system for the analysis of adaptive plasticity in the spinal cord.

## Supporting Information

S1 Fig Reconstruction and post section alignment of spinal cords. (A) Reconstruction of the spinal cord sections using the Reconstruct software[23]. Serial section images were aligned by means of 4 optical reference points and individual motoneurons were traced. (B and C) Representation of the motor pools before and after post-section alignment, which was performed in order to correct for the physiologically increased diameter of spinal sections at the level of the cervical enlargement.
(EPS)

S2 Fig Behavioral improvement due to enriched environment housing starting at birth. (A) Since wt animals are already at a very high performance level, enriched environment housing is not able to induce further improvement of their motor coordination (NH: 11.74 ± 0.86 s, N = 14; EE: 10.37 ± 0.76 s, N = 10; p = 0.26). (B) In contrast, already at the age of 4 weeks *Sema3F* mutants show a clear improvement of their motor performance on the grid walk test due to enriched environment housing starting at birth (NH: 25.58 ± 2.56 s, N = 12; EE: 16.74 ± 1.71, N = 9; p = 0.0087).
(EPS)

S3 Fig Enriched environment housing does not cause changes in neuromuscular junctions of the ventral forelimb. The surface area of neuromuscular junctions in the ventral forelimb is comparable (p = 0.11, one-way ANOVA) between *Sema3F* animals after normal housing (564.4 ± 16.6 μm^2^ vs. 514.5 ± 14.3 μm^2^, N = 3) or enriched environment housing starting at birth (480.4 ± 27.0 μm^2^ vs. 524.6 ± 22.0 μm^2^, N = 3). Scale bar 20 μm.
(EPS)

S4 Fig Correlation of the Scatter Index and motor performance of *Sema3F* animals. The analysis of individual animals revealed a direct correlation between the performance in the grid walk test and the scatter index of the ventrally projecting motor pool (slope: 0.00008098 ± 0.00003246; derivation from zero: p = 0.03).
(EPS)

S5 Fig Formation of PNNs during early postnatal development. (A-C) In early postnatal development no significant differences in the formation of PNNs on retrogradely traced motor neurons were found at P21 (none: average 80.0%, p = 0.78; weak: average 16.9%, p = 0.78; strong: average 3.1%, p = 0.53), P14 (none: average 93.4%, p = 0.74; weak: average 5.8%, p = 0.73; strong: average 0.9%, p = 0.69) or P7 (none: average 95.72%, p = 0.56; weak: average 3.54%, p = 0.34; strong: average 0.73%, p = 0.82). Statistical analysis: N = 3 for each group.
(EPS)
